# A Rare Cause of Female Urinary Retention: Large Urethral Stone Leading to Bilateral Hydronephrosis

**DOI:** 10.7759/cureus.89759

**Published:** 2025-08-10

**Authors:** Fadhel Yusuf, Amr Elmekresh, Thureya Binashour, Hamzeh Esmaeilpour, Senthil Kumar, Mohamed Kaya, Yaser Saeedi

**Affiliations:** 1 Urology, Dubai Hospital, Dubai, ARE; 2 Urology, Dubai Health, Dubai, ARE

**Keywords:** dysuria, female urinary retention, obstructive hydronephrosis, obstructive uropathy, urethral stone

## Abstract

Urethral calculi are an uncommon clinical finding in females due to their relatively short and wide urethra, which typically facilitates the spontaneous passage of stones without impaction. We present a unique case of a 62-year-old female with no prior medical or surgical history who developed acute urinary retention lasting seven hours. She reported suprapubic discomfort but denied fever or flank pain. Examination revealed a distended bladder, and laboratory investigations, including renal function and inflammatory markers, were normal.

Computed tomography kidneys, ureter, bladder (CT KUB) revealed a markedly distended bladder, bilateral moderate hydronephrosis, and a 2.5-cm longitudinal stone within the urethra. No renal or ureteric stones were identified. A Foley catheter was inserted successfully, draining urine and relieving symptoms, and the patient was discharged with the catheter in place for follow-up.

She presented a few days later after accidental dislodgement of the Foley catheter at home. Examination revealed a portion of the stone protruding from the urethral meatus. Under appropriate analgesia and sterile technique, the entire stone was successfully removed in one piece using forceps. The patient remained stable and was monitored for post-obstructive diuresis.

This case illustrates a rare presentation of a large female urethral stone causing acute urinary retention and bilateral hydronephrosis. It also highlights the potential for spontaneous expulsion and the importance of considering urethral stones in female patients with urinary retention. Due to the rarity of such presentations, this case adds valuable insight to the limited literature on urethral stones in women.

## Introduction

Urolithiasis is a common condition, with urinary tract calculi typically forming in the kidneys and migrating distally. However, the presence of urethral calculi, particularly in females, is exceedingly rare, accounting for less than 1% of all urinary tract stones [1]. In contrast to males, whose longer and more complex urethral anatomy may predispose to stone impaction or formation, females possess a short and straight urethra, which generally facilitates spontaneous passage of small stones [2,3]. Most urethral stones in females are secondary to either migration from the upper urinary tract or underlying anatomic abnormalities such as urethral diverticula, strictures, or foreign bodies [4].

Female urethral stones can present with non-specific lower urinary tract symptoms, including dysuria, urinary retention, hematuria, or pelvic pain [5]. Acute urinary retention caused by a large obstructive urethral stone is particularly uncommon, and bilateral hydronephrosis as a secondary complication is even more exceptional. Cross-sectional imaging, particularly computed tomography (CT), plays a crucial role in identifying the size, location, and associated complications of urethral calculi [6].

Herein, we present a rare case of a 62-year-old female with acute urinary retention caused by a giant urethral stone, complicated by bilateral hydronephrosis. The stone eventually became externally visible and was removed intact at the bedside. This case highlights the diagnostic challenges, clinical implications, and management strategies for urethral calculi in females and adds to the limited literature on this rare entity.

## Case presentation

A 62-year-old female with no prior medical or surgical history presented to another facility with acute urinary retention lasting seven hours. She reported suprapubic pain but denied any flank pain, fever, or constitutional symptoms. Physical examination revealed a distended bladder with associated suprapubic tenderness, but no flank or costovertebral angle tenderness was noted. Her vital signs were stable, and laboratory investigations, including inflammatory markers and renal function tests, were within normal limits, with a creatinine level of 0.81 mg/dL.

**Figure 1:**
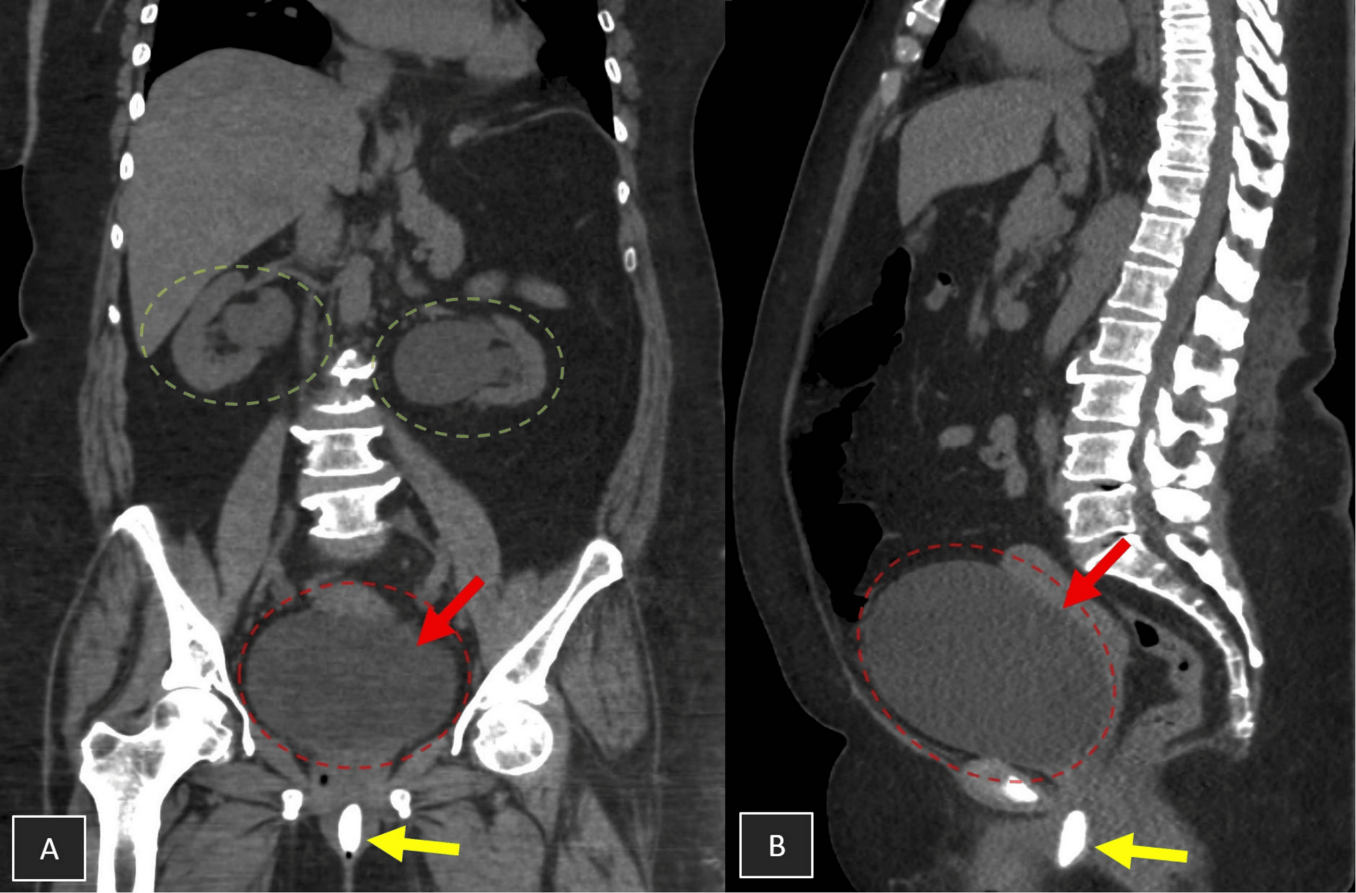
CT KUB showing bilateral hydronephrosis and a distended bladder due to an obstructive urethral stone. A: Coronal view – showing bilateral hydronephrosis (green circles), a distended bladder (red arrow and circle), and a urethral stone (yellow arrow). B: Sagittal view – showing a distended bladder (red arrow and circle) and a urethral stone (yellow arrow). CT KUB: computed tomography of the kidneys, ureters, and bladder

A non-contrast computed tomography of the kidneys, ureters, and bladder (CT KUB) revealed a significantly distended urinary bladder, bilateral moderate hydronephrosis, and dilated ureters. Notably, a 2.5-cm linear calcification was visualized within the urethra, consistent with a urethral stone (Figure 1). No renal or ureteric calculi were identified, and an incidental hiatus hernia was also noted.

A urethral catheter was inserted without difficulty, resulting in immediate decompression of the bladder. It was presumed that the urethral stone was displaced into the bladder during catheter insertion. The patient was discharged home with a Foley catheter in place and scheduled for follow-up at our urology clinic for possible stone removal pending anesthesia fitness.

**Figure 2:**
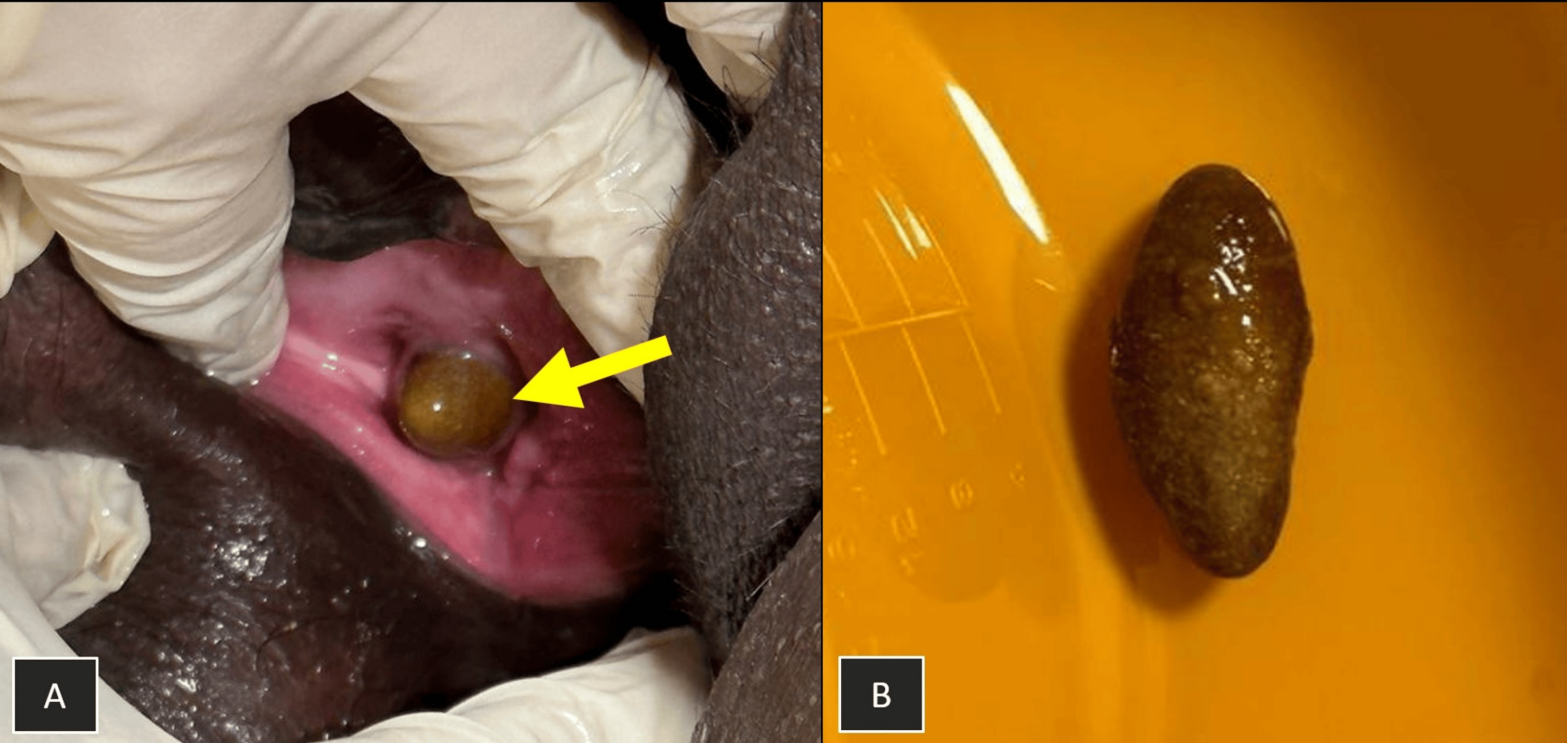
Large urethral stone A: Large stone protruding from the urethral meatus (arrow). B: The urethral stone following intact removal using sterile forceps.

The patient presented to our hospital a few days later following accidental dislodgement of the Foley catheter at home. She reported urinary leakage and discomfort. It was presumed that the catheter balloon had ruptured due to contact with the stone, causing the catheter to slip out. On examination, part of a large stone was visible protruding from the urethral meatus (Figure 2a). Under appropriate analgesia and sterile conditions, the stone was removed intact using forceps (Figure 2b).

The patient was closely observed for several hours after the procedure to monitor for post-obstructive diuresis. She remained stable throughout and was subsequently discharged. The retrieved stone, which measured approximately 2.5 cm, was retained by the family and not sent for laboratory analysis.

This case highlights the rarity of urethral calculi in females, a condition more commonly encountered in males. Its presentation with bilateral hydronephrosis and retention due to a large impacted stone is unusual, and spontaneous protrusion allowing bedside removal is particularly rare. Given the limited literature on female urethral stones, especially of this size, we present this case to add to the existing body of evidence.

## Discussion

Female urethral stones remain a seldom-encountered clinical diagnosis. Their incidence is low due to the short length and wider caliber of the female urethra, which usually enables the passage of calculi without impaction [3]. In most reported cases, urethral stones are either secondary to migration from the upper tract or develop in situ due to underlying pathology such as chronic infections, urethral diverticulum, or foreign bodies [4].

The large size of the calculus (2.5 cm) makes spontaneous migration through the urethra unlikely, which may explain the significant obstruction and resulting bilateral hydronephrosis observed on CT. To our knowledge, there are very few documented cases in the literature reporting urethral calculi of this size in females leading to upper urinary tract dilation [7].

Urinary retention in females is itself a rare phenomenon, comprising less than 5% of emergency urological consultations in women [8]. When it does occur, common causes include obstructive masses, neurogenic bladder dysfunction, or postoperative complications. Urethral stones are an uncommon but important addition to the differential diagnosis, especially in older women or those presenting with a history of lower urinary tract symptoms refractory to initial treatment.

Diagnosis relies on a combination of physical examination and imaging. In this case, the initial presentation involved suprapubic tenderness with a palpable bladder, and imaging via CT KUB provided critical information regarding the presence of bilateral hydronephrosis and the location of the urethral calculus. Ultrasound may have limited utility in identifying urethral stones, particularly when impacted near the meatus [9]. While cystoscopy is the gold standard for direct visualization and management, CT remains highly sensitive in visualizing urethral and bladder stones and assessing complications [6].

Management depends on the size, location, symptoms, and associated complications. Small stones may pass spontaneously or be expressed manually. Larger or impacted stones typically require endoscopic extraction or lithotripsy [10]. In our case, the stone had migrated distally to the point of partial extrusion from the urethral meatus, allowing for removal under analgesia with sterile forceps. Conservative management was successful, and the patient remained stable with no post-obstructive diuresis. However, larger stones retained within the urethra may pose risks such as pressure necrosis, urethral rupture, or persistent infection, necessitating prompt intervention.

## Conclusions

This case highlights a rare presentation of a large urethral stone in a female patient, causing acute urinary retention and bilateral hydronephrosis, an uncommon but clinically important scenario. The spontaneous migration of the stone to the urethral meatus and its successful bedside extraction emphasize the value of thorough clinical evaluation, CT imaging, and individualized management.

Although urethral calculi are rare in females, they should be considered in cases of unexplained lower urinary tract obstruction, particularly when upper urinary tract changes are present. Early identification and intervention can prevent complications and reduce the need for invasive procedures. By sharing this case, we aim to contribute to the limited literature on female urethral stones, underline the importance of maintaining a broad differential diagnosis, and encourage further reporting to enhance clinical awareness and guide future diagnostic and therapeutic strategies.
